# Reducing ethnic inequalities in experiences and outcomes of mental illness: A realist informed national programme evaluation

**DOI:** 10.1371/journal.pmen.0000643

**Published:** 2026-06-29

**Authors:** Kamaldeep Bhui, Phuong Hua, Sarah-Jane Fenton, Roisin Mooney

**Affiliations:** 1 CHIMES Collaborative, Academic Department of Psychiatry, Wadham College, University of Oxford, Oxford, United Kingdom; 2 Nuffield Department of Primary Care Health Sciences, University of Oxford, Oxford, United Kingdom; 3 Health Services Management Centre, University of Birmingham, Birmingham, United Kingdom; 4 CHIMES Collaborative, Academic Department of Psychiatry, University of Oxford, Oxford, United Kingdom; PLOS: Public Library of Science, UNITED KINGDOM OF GREAT BRITAIN AND NORTHERN IRELAND

## Abstract

Black and minority ethnic communities are more likely to receive coercive mental health care, and involuntary admissions via crisis and criminal justice agencies. Lived experience informed interventions may be better able to address structural determinants, overcome resistance, and motivate innovations. The Synergia Collaborative Centre (SCC) sought to drive systems reform through insights from lived experience data, evidence syntheses, co-design and creative communications.We report a qualitative study of the SCC, applying a realist-informed secondary analysis method to interview data from an independent evaluation. Twenty-four stakeholders’ transcripts were analysed alongside minutes of operational and advisory board meetings and project documents to discern how SCC was received by different stakeholders and how and whether it operated. Context-Mechanism-Outcome (CMO) configurations were generated drawing on ecological systems theory to enhance understanding of context, therefore, the findings were organised by eco-social levels. The programme successfully built confidence and leadership capacity, centring on lived experience, co-design and embedding local evidence-based actions through creative communications to motivate and engage partners. Power imbalances between established experts and new leaders risked disengagement of both; pessimism was born of historical failures of similar programmes and a lack of sustained resources; promoting policy makers and professional participants in the change process, alongside local community stakeholders were proposed for future traction. We distil facilitators and barriers to progressive change in community, health, and social systems as observed by stakeholders and as reported in their interviews as part of an evaluation of SCC.This research generated a refined programme theory exploring how the programme worked, to inform and generate recommendations for future programmes. We discuss strengths and limitations of using realist-informed secondary analysis methods for post-hoc assessment of complex programmes.

## Introduction

Evidence from North America, Europe, and the UK shows ethnic minorities and migrants are most likely to experience involuntarily care, on the grounds of lacking insight or the risks of harms to themselves or others [1–5]. Involuntary care in ethnic minorities and migrants follows relatively more crises, emergency room attendance, and contact with the police, criminal justice and forensic systems [1,6,7]. Compared with White majority patients, ethnic minority groups are more likely to experience longer hospital stays and have limited access to psychotherapeutic services [8,9].

Urban environments may explain higher rates of involuntary care, perhaps related to a greater burden of social determinants of mental illness, as well as poor housing, lack of social support, fear of crime and fear for personal safety. Importantly, more community mental health professionals, better housing support, and psychological therapies are associated with lower risks of involuntary care, although much of the overall variance remains unexplained [10,11]. Legislative changes and austerity measures can both culminate in more involuntary admissions [12].

Race disparities in mental health care are an additional cost burden and savings from reducing these could be usefully invested in improving community solutions and non-coercive pathways to care [13,14]. The COVID-19 pandemic further highlighted the importance of social and intersectional vulnerabilities to poor health and the role of structural factors in unequal health outcomes.

*Inside Outside* (2003) was the first national strategy that aimed to reduce ethnic inequalities in mental health care experiences and outcomes in England [15]. It was the official government response to the inquiry into the death of David Rocky Bennett [16,17]. The proposed actions included building cultural capability, deploying community development workers, and implementing practical measures to reduce coercion and poor care. This was followed by *Delivering Race Equality in Mental Health Care Action Plan* (DRE, 2005), which aspired to reduce ethnic disparities in mental health care by 2010 [18,19]. During this time, national data on involuntary admissions were reviewed. There was slow and steady progress towards building better pathways to care, partnerships with the voluntary sector, and workforce training on race equality and cultural competency [18,19]. Cycles of transformational leadership were essential, moving from ideological aspirations to practical actions and back again [20–22]. However, the programme evaluation showed that the tasks of developing and embedding innovations and improving best practice were underestimated. Some regions abandoned the plan due to limited budgets and competing priorities [18,19]. There were also objections to using terms such as ‘institutional racism’ on grounds that this reinforced race-based solutions rather than highlight cultural and systems change in training and practice [23]. Despite extensive monitoring of detentions by ethnicity, there was no material reduction in admission and detention rates among ethnic minority groups [19]. The lack of progress, pessimism, disputes about the evidence, insistence on not naming racism in policy [19,23] all led to a period of *dejected silence* by communities and practitioners and policy makers. Termination of the programme was felt as neglectful of lived experience experts (and professionals) who had invested so much, hoping their contributions would help tackle ethnic inequalities [24].

It was during this period of dejected silence, before the pandemic, that community activists began to highlight the persistence of race and ethnic disparities [25]. Furthermore, the financial crisis at the time placed pressure on mental health systems, resulting in a rise in detentions and fragmentation of community service models. To respond to this context, the Synergi Collaborative Centre (SCC) was commissioned in 2017 (see https://legacy.synergicollaborativecentre.co.uk/about-us/) as an independent knowledge hub and centre of excellence to understand and address the systems drivers of ethnic inequalities in the experience and outcomes of severe mental illness.

Given the necessarily non-linear approach to complex systems change and the involvement of multiple partners, the programme components were not pre-defined; rather these emerged during conversations within a series of ‘creative space’ events. New solutions were to be co-designed by a diverse range of key stakeholders in statutory and non-statutory sectors. Lived experiences informed the participatory co-design processes and motivated the adoption of cross-sectoral coalitions and actions [26].

The initial SSC Theory of Change (ToC, see Table 1, Appendix A in S1 Text) aligned with the *COM-B* model of behavioural change that seeks to improve *capabilities*, use *opportunities*, and *motivate* people to act [27]. The TOC acknowledged previous initiatives and policies (Context) and sought to democratise knowledge creation, assemble multiple and profound accounts of lived experiences, improve health literacy, and deploy creative communications [28] (noted as potential Mechanisms to build capabilities and motivate stakeholders to act), all to inform progressive policy and practice. This, we anticipated, would reduce structural racism in mental health systems and improve mental health status of those using services (Outcomes). However, given the complex systems in which the programme was to be located, there could be many outcomes that fell short of actual changes in mental health care of patients or reduction in inequalities. We wished to capture these mid-range impacts as this could inform more effective design of future programmes.

**Table 1:** Plan for SCC programme management, sharing scientific knowledge, and communications based on the SCC’s Theory of Change (ToC).

	Programme management	Assembling and Sharing Scientific Knowledge	Communications and Media
**Tasks/Aims**	Efficient programme management. Develop new collaborative ways of working with diverse interests and ideologies. Generate new ways of doing research and finding new solutions.	Broaden the expert knowledge base by foregrounding lived experience, co-production and participatory processes. Use best methods for a critical evidence base. Place critical evidence alongside experience data for maximum impact.	Make knowledge accessible through arts, blogs, performance, and accessible summaries.
**Methods and products by which change would happen**	Annual reports; advisory board reports and meetings; grant applications; stakeholder insight report; weekly director meetings; monthly project team meetings.	Produce briefing papers; academic publications; photovoice workshops; audio-documentary (podcast); big dataset analysis; national priority setting survey; tool development and dissemination based on stakeholder insight reports; Participatory Action Research; evidence reviews, lived experience research. Build consensus amongst senior leaders and policy makers on what is needed.	Public engagement and impact – newsletters; podcasts; photovoice artefacts; blog; national campaign and pledge; presentations at community groups; creative spaces events. Motivate changes in policy, commissioning, and practice. Enable communities to take power and launch local actions and campaigns.

### Aims

This study aimed to:

1) Conduct a post-hoc assessment of SCC using data from an independent evaluation which interviewed stakeholders who had contact with the SCC.
2) Identify critical learning alongside contextual factors, mechanisms and outcomes that influenced stakeholders’ experiences and generated some of the consequent outcomes.
3) Develop a programme level theory that could inform how such programmes might be organised and delivered in the future.

## Methods

### Ethics statement

A favourable ethics review was received from the Queen Mary University of London Research Ethics Committee (QMERC2019/73) in December 2023. This permitted re-analysis of the interview data, collected as part of an independent evaluation that took place from 01/02/21 to 31/07/21.

### Theoretical approach

We wanted to understand how the programme operated according to stakeholders’ experiences, that had been gathered as part of an independent evaluation. We sought to value stakeholders’ views as real world accounts (aligned with realist perspectives on experiential data), that were captured whilst the programme was operating – but to view these anew through a realist-informed secondary analysis to understand on a broader level what outcomes SCC had generated for whom, in what circumstances and why. Realist philosophy posits that the success or failure of an intervention is determined by individuals in the system making contextualised decisions in response to the intervention [29]. These decisions are influenced by the individual’s values, beliefs, norms, previous experiences and circumstances of the time; different contexts may drive different mechanisms which can change the outcome [30].

A realist-informed secondary analysis approach [31] was underpinned by ecological systems theory [32] in order to understand the impact of context (local, national, global) on the activation of generative mechanisms and outcomes of the programme. We did not adopt this design uncritically. Although realist evaluations are usually only applied during primary realist evaluations, we undertook a realist-informed *secondary* analysis. This was efficient in terms of using existing data and minimising cost, as we were not resourced to undertake a fuller set of interviews which were originally planned. Furthermore, the COVID-19 pandemic also meant we could not progress ethics approval or an evaluation in a reasonable time frame as we approached the end of the SCC programme. As evaluation data were already collected, this made for an efficient process making best use of these data. Many programmes such as this are never evaluated due the complexity and practical challenges that often arise. We wished to not be silent on the challenges and obstacles, as well as assess value, to inform future programmes.

We followed the RAMESES (Realist And MEta-narrative Evidence Syntheses: Evolving Standards of evidence synthesis) in reporting the results of the study [33].

**Fig 1:**
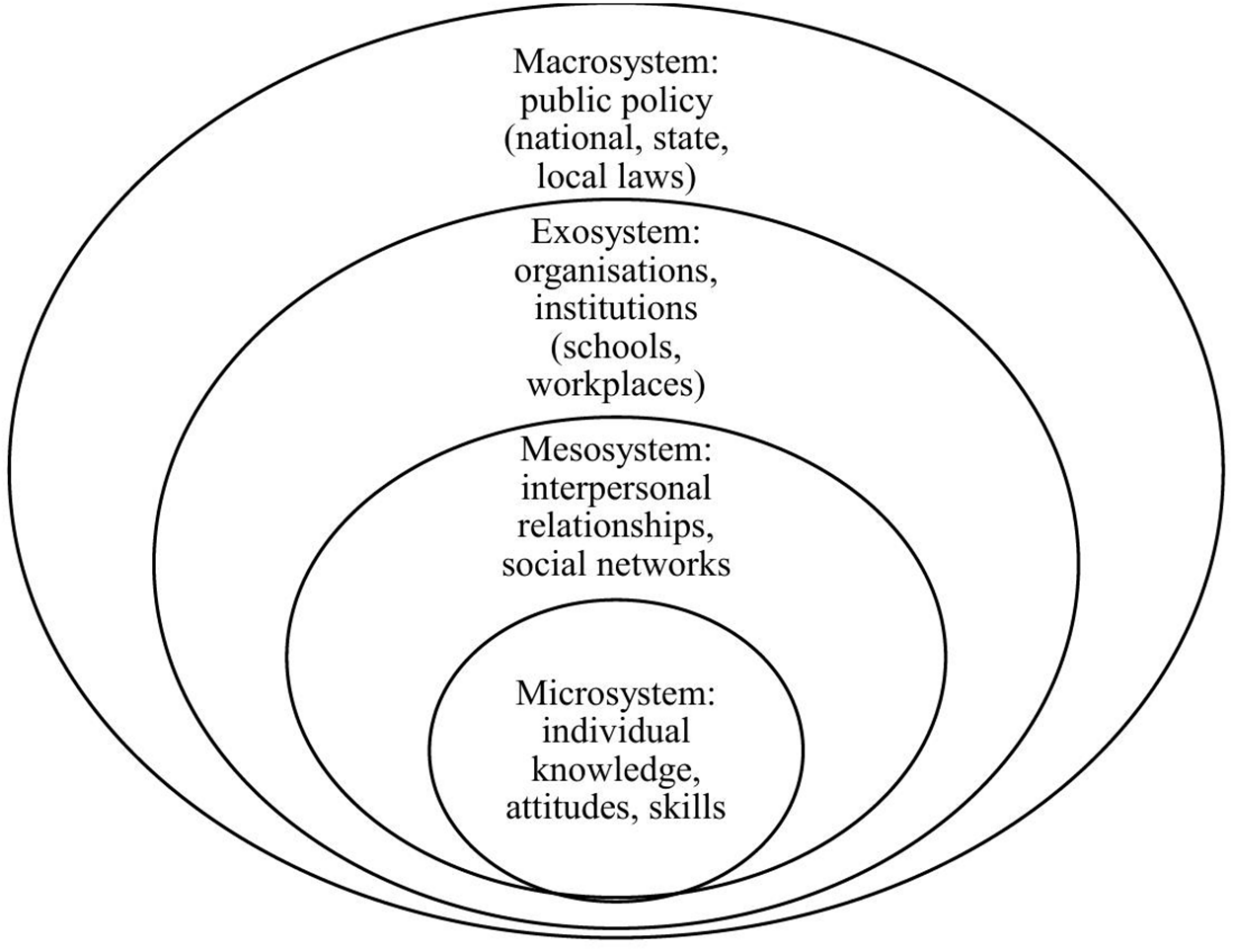
Socioecological model.

Given the multiple influences on mental health and the nature of health and social care systems, we applied a Socioecological Model (SEM, see Fig 1) drawing on ecological systems theory to organise the findings. The SEM recognises multiple inter-related layers of a person’s environment and impacts on behaviours [32]. The *microsystem* refers to the immediate environment including home or workplace. The *mesosystem* relates to inter-personal relationships and social networks. The *exosystem* refers to social structures and institutions (e.g., health, social, employment and faith-based) that impinge upon people’s lives. Finally, the outer layer is the *macrosystem*, where legislation, policy and wider macro issues that affect a society reside [32]. SEM has informed many public health and mental health policies [34]. SCC may have had different impacts or been impacted by different events shaping stakeholders reactions at each of these levels (i.e., Black Lives Matter or COVID-19 at the macro level may influence stakeholder perception or engagement). We wished to be sure of capturing these, where the findings were compelling and sufficiently supported in the analysis across participants. During the analysis, we relied on judgements of validity, importance, and plausibility, as well value in iterating or replacing the original theory of change if warranted.

### Data sources

We used interview transcripts from an independent evaluation centred around stakeholders’ experience of the SCC. The original interviews for the independent evaluation were not considered research interviews, as they were commissioned as consultancy work. Out of 44 people who were interviewed 27 agreed to share their transcripts with SCC. As it was unclear what the purpose of sharing the transcripts was, we contacted those who agreed for their transcript to be shared to confirm that they were happy for their transcript to be included in our secondary data analysis. This required amendments to an ethics application that was originally submitted for collecting new data by interview; this was amended to include re-analysis of the original transcripts. The closure of all non-COVID-19 studies at the time also meant the ethics application was delayed significantly. Out of the 27, 24 agreed (by email; see professional roles of stakeholders in Appendix B in S1 Text) and 3 were uncontactable. We did not have information on those who did not consent. We were not permitted, as is usual with ethical approval, to follow up and inquire why they did not wish to participate or indeed make use of any of their data that might be held by the independent evaluation team.

**Fig 2:**
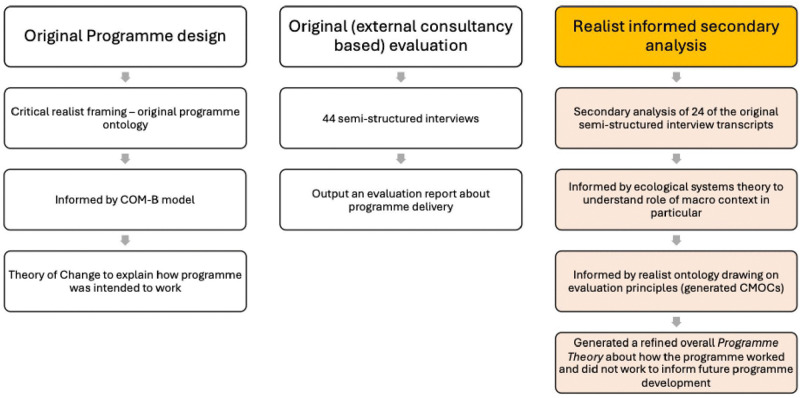
Theoretical orientation, data sources and approach.

The average duration of original interviews was 30 minutes. The funding application, the initial Theory of Change, and the transcripts, were analysed alongside minutes of the quarterly Advisory Board Meetings, which were held between 2017–2021. The Independent Advisory Board comprised a diverse group of academics, mental health experts, providers, commissioners and executives from charity and policy organisations (see Fig 2 for data sources and approach).

### Data analysis

The transcripts for all stakeholder interviews and Advisory Board minutes were analysed using qualitative software (MAXQDA 2022, Release 22.2.1). An iterative three step process of analysis was followed:

**Step 1:** The interviews were initially thematically analysed to develop key themes using a realist-informed approach based on guidelines [35,36], and using retroduction, which involves the researcher moving backwards and forwards between what is noted in the interviews and a priori theories or hypotheses to understand what is producing the outcomes [31,37]. As part of retroduction, the analysis used ‘three ears of listening’ an approach established within a realist evaluation to account for what was said explicitly (Ear 1), what was implied (Ear 2) and what was unsaid or ‘felt’ as an observation rather than expressed directly (Ear 3) [38]. Aligned with qualitative methods, this required significant reflexivity and discussion of the emergent findings between the research team to be assured of validity. From this initial stage, we generated a Context, Mechanisms and Outcomes (CMO) realist-informed coding framework that helped generate CMO configurations to organise the core findings and concepts from the interview data. The CMO referred to:
  - Context – factors, events or stressors at all levels of the SEM (including health inequalities) that could have influenced the stakeholders’ experiences of the SCC.
  - Mechanisms – Reasoning refers to internal psychological processes that captured cognitive mechanisms (e.g., use of logic), values, emotions, and a combination of these processes [39]. Resources refers to external opportunities that were available to stakeholders within their roles to mitigate the effects of contextual moderators.
  - Outcomes – impacts on the stakeholder and their experiences of impact of SCC.
**Step 2:** Advisory Board minutes were analysed thematically and coded using a CMO framework (Appendix C in S1 Text). These were used to cross-validate the findings from stakeholder interviews.
**Step 3:** A process of synthesis across these two types of data took place. In line with realist-informed methodology, the findings of this evaluation were articulated as *demi-regularities* [40] – recurring but contingent patterns of stakeholder reasoning and response – expressed as both positive and negative contrastives. This approach draws on the retroductive theorising framework [40], which emphasises identifying generative mechanisms by moving iteratively between empirical data and theory. Outcomes are understood not in isolation but through comparison, for example, why an outcome occurred in one case but not another despite similar conditions. Adopting this principle, we presented stakeholder responses as *+ve (positive) or -ve (negative) contrastives* to make visible the mechanisms that shaped perceived success or limitation of the SCC. This framing allowed us to infer mechanisms that were generative of specific outcomes, by comparing polarised views. We integrated our CMO codes and thematic findings towards an iterated programme theory to try and explain how or why elements of the programme were successful or not in particular contexts and which mechanisms were triggered. The data were retrospectively analysed, and we did not observe behaviours directly, therefore, inferences were drawn by the research team during the analysis based on the expressed experiences and from direct quotations of the stakeholders.

## Results

The CMO configurations captured positive and critical views on whether SCC was meeting its original objectives and what experience stakeholders had of SCC.

### Positive CMOs (see Fig 3, Appendix C in S1 Text)

*Macrosystem:* The Covid-19 pandemic and Black Lives Matter protests exposed racial tensions and distrust from ethnic minority communities (Context). Thus, it was viewed as a great achievement that SCC was established in advance of this period of enhanced societal awareness of racial tensions (Context). This was repeatedly interpreted as evidence of strategic foresight and SCC’s role as a pioneer in racial justice and mental health; this was also linked to greater population awareness (Reasoning - Mechanism). Funding (Resources – Mechanism) for SCC was obtained during a period of less public awareness about addressing racial inequalities in healthcare as a priority area, meaning the formation of SCC itself was timely.

**Fig 3:**
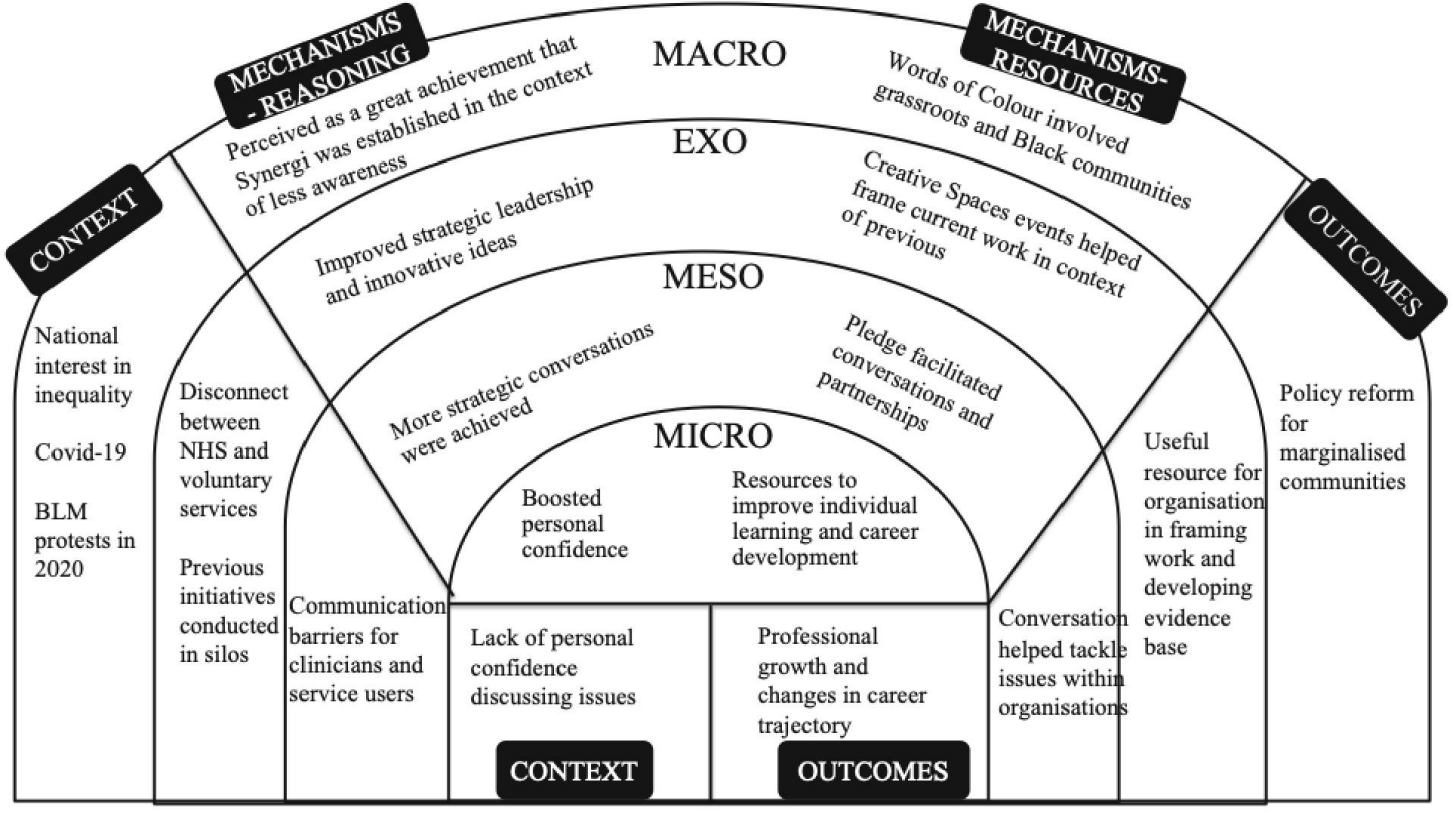
CMOs at each ecosystem level: positive views.

*Exosystem:* Already established connections between services in the statutory and non-statutory sectors (Context) were identified as potential contributors to the slow systemic change (Mechanism) and thus undermining impacts of SCC (Outcome). SCC was praised for facilitating collaborations between the sectors (Mechanism) through networking events (e.g., Creative Spaces, see https://legacy.synergicollaborativecentre.co.uk/connect/creative-spaces/) that brought some groups together in collaborative and innovative spaces (Mechanism of resources) around a central purpose (Mechanism of reasoning). This connected equalities initiatives and improved support strategies for marginalised groups (Outcome).

*Mesosystem*: Stakeholder interviews proposed persistent barriers to conversations about health inequalities and challenges for developing strategic partnerships with stakeholders (Context). SCC facilitated actors to engage in more strategic conversations (Mechanism - reasoning), which led to building of stronger partnerships and organisational identities (Outcome). This pattern suggests a demi-regularly whereby SCC embedded equity dialogues in strategic planning of partner organisations.

*Microsystem*: Stakeholders who reported a lack of knowledge about how to engage in discussions about race (Context) reported a boost in confidence to engage in sensitive conversations (Mechanism - reasoning) to improve literacy (Outcomes). This reinforced capacity-building (Mechanism – resources), engagement with equity work and professional growth that enabled individuals to develop skills that facilitated collaboration (Outcome).

SCC was viewed as a learning space producing good quality and rapid research.

*I didn’t feel confident in talking about issues, knowing what language …. knowing what the right words were … they sort of gave me the confidence to have the language, talk about it (Health Improvement Principal, Public Health, City Council)*

SCC was regarded as a figurative *relationship counsellor* for stakeholders across all sectors. Stakeholders from community organisations had previous experiences of forming partnerships that were not robust or sustained (Context); involvement allowed them to evaluate progress following participation in SCC (Mechanism). There were perceptions that SCC improved their organisation’s image as a strategic partner (Outcome).

*…having SCC, as a partner, and having the Pledge has enabled the (mental health) trust to take us much more seriously, as a strategic partner, not just someone who they can ask to do little consultations here…helping us kind of facilitate a dialogue (Director, Community organisation).*

Stakeholders from NHS trusts also perceived that relationships with voluntary services were strengthened through SCC’s catalytic role in fostering relationships.

*I learned a lot about …the perception of voluntary services. It seen from the…lens in terms of the NHS. And I think that relationship was developing but was happening at a slower pace…SCC has actually propelled that a little bit, having a relationship counsellor…(Inclusion Lead, NHS Trust)*

NHS organisations involved in SCC were large and diverse (Context) which made it a significant achievement that SCC was able to promote organisational commitment (Mechanism of building trusting relationships between stakeholders) towards a joint statement of intent (Outcome).

*I think working with some of our partners…we’ve been able to create a systematic review and get to a statement of intent for the whole organisation to work to adopt, I think was phenomenal really, and not underestimate the challenges that he is with such a big system. (Police Services for Mental Health)*

### Negative CMOs (see Fig 4, Appendix C in S1 Text)

*Macrosystem:* Across interviews, stakeholders consistently identified that policy and practice change were highlighted and accelerated following global events such as Black Lives Matter and Covid-19, but this was still too slow. Participants said that this highlighted racial inequalities, in relation to systemic racism and health (Context).The slower pace of change observed by stakeholders was reported to contribute to scepticism (Mechanism - reasoning) about the sustainability of racial equity programmes and SCC’s legacy, given awareness of the outcomes from previous programme and the absence of long-term structural reform or funding (historical pessimism). Indeed, mostly previous programmes were short-lasting and insufficiently resourced nor well evaluated, which meant judgements about value and impact were always based on opinion rather than data. Pessimism therefore impacted engagement and trust building (Mechanisms – resources) due to the perception that SCC was unlikely to have sustainable or lasting impacts (Mechanism). This suggests that in contexts where there is lack of continuity of race equity initiatives, stakeholders may become disillusioned (Outcome), undermining progression.

**Fig 4:**
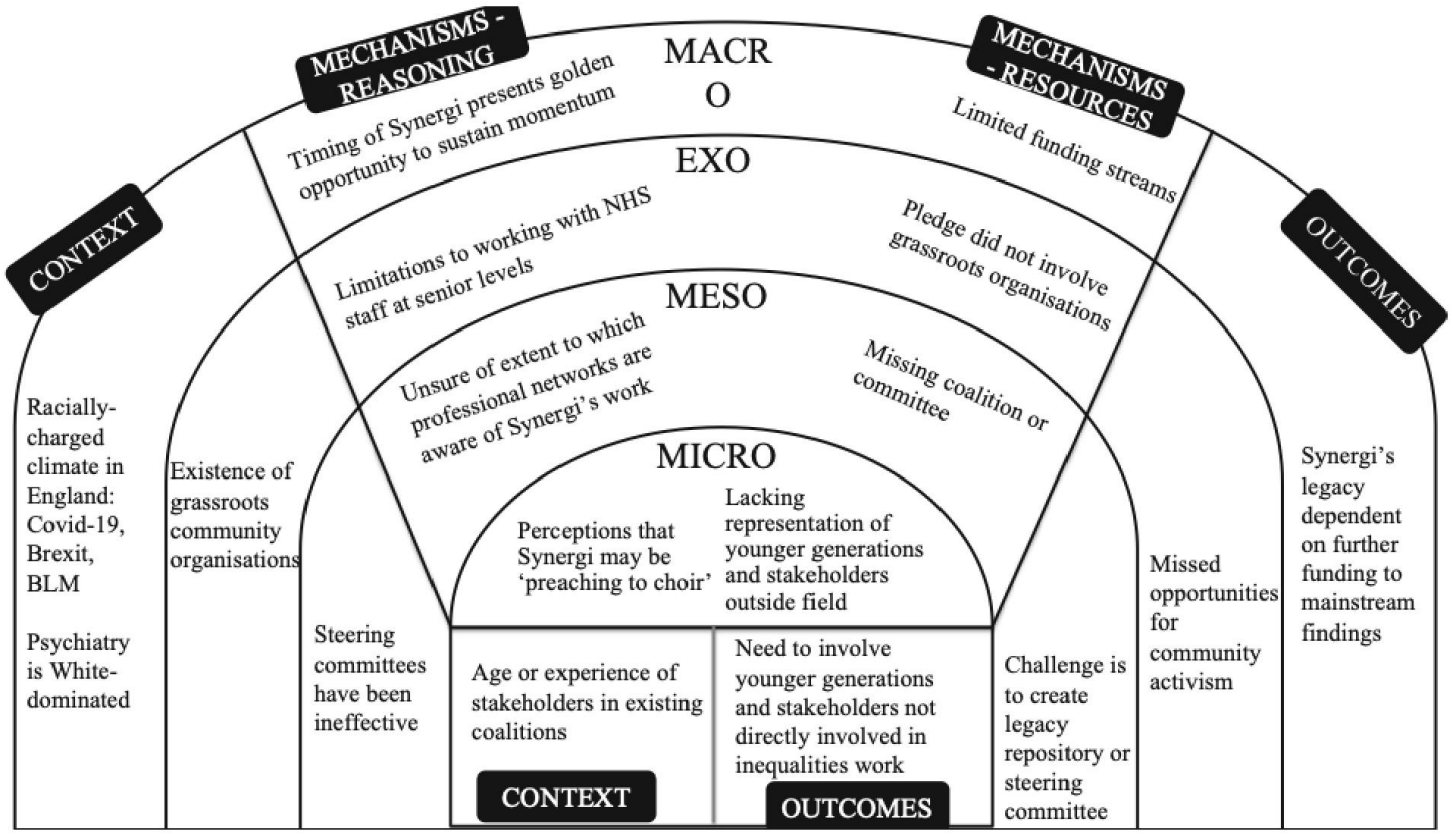
CMOs at each ecosystem level: negative view.

*Exosystem:* There was repeated reference to the UK’s history of short-term funded initiatives prior to contemporary programmes such as the Race and Health Observatory, Black Thrive (a recently formed NGO), as well as SCC (Context). This insecure funding model influenced how programmes develop, how they are perceived and received. This perhaps explains consistent perceptions that SCC, like previous initiatives, would be transient and under-resourced (Mechanism – Reasoning). When initiatives lack secure funding, they trigger mechanisms of doubt and disinterest, which limit engagement and undermine commitment and motivation. This undermines SCC’s perceived impact and participants’ trust of sustained institutional investment. This might lead to negative evaluation of the role of SCC, as well as disengagement so making SCC less effective (Outcome)

Additionally, stakeholders expressed frustration about missed opportunities to engage grassroots organisations (Reasoning), even though SCC was involved with many. This may reflect tendencies to consider organisations with established relationships and aligned plans as more important than critical ones or new ones with a different approach and vision. One risk to future programmes is how community-based experts or perceived leaders can become defensive and protect silos of expertise, leading to mechanisms of exclusion and fragmentation. This also undermines cross-sector collaboration and coalitions thus undermining the potential impact of programmes (Outcome).

*Mesosystem:* When past experiences (i.e., including systemic or institutional racism) reduced trust, initiatives need to establish stronger coalitions to activate mechanisms of relational trust in continuity. Thus, there were perceptions that SCC lacked sufficient presence among some professional networks (Mechanism) and need to establish a steering coalition and without this, existing networks would weaken (Outcome). This view contradicted the recommendation to be more community focussed on local organisations, and not professional networks.

*Microsystem:* Among stakeholders with long-standing involvement in racial justice work (Context), there was a recurring sense that their hard-won experience was undervalued (Reasoning). This demi-regularity reflects a mechanism of disengagement which subsequently reduces opportunities for interdisciplinary learning, innovation and fresh perspectives (Outcome). Active mechanisms at the microsystem level related to stakeholders’ existing roles (increased agency) and previous experiences in mental health and inequality (praxis). This meant they were already familiar with the issues (increased knowledge or awareness) (Context). When this came into conflict within the programme desire to reduce power differences and centre around lived experience, it resulted in disengagement (Mechanism) of experts who felt slowed down; this finding may well explain why programmes fail to include lived experience or form silos around specific charismatic leaders and particular ideologies of how to solve a problem. Thus, praxis knowledge was felt to be undervalued (Outcome). This was a barrier to SCC’s progress moving forward as it hindered learning between more and less experienced actors, and undermined respect for different types of knowledge (Outcomes).

A shared sentiment among stakeholders, particularly NHS and community organisations, was that SCC’s legacy was yet to be established, and progress required longer term commissioning and investment, which was called for (Outcome).

*I think that’s the thing about, you know projects like this…timespan two to three years, four years if you’re lucky, but then once that work comes to an end. Everybody Pat’s himself on the back. We’ve delivered on this, and then they walk away…That’s probably some of these funding streams…It would be great to see some kind of going forward investment in that…so does not fall on stony ground. (Community Development Manager, Community organisation)*

A clinical psychologist recounted that the beginning of their role coincided with the era of the DRE (Context) which they observed did not have a lasting impact. This shaped their views that further work was required from SCC (Mechanism) to actively preserve the history of work and ensure it is public knowledge.

*One thing that SCC, or any other organization involved in this could usefully do is create something around history, synthesizing all of these things that have gone before. I first started working in this specific role during the era of delivering race equality and mental illness…as soon as it was over everything disappeared.*

The stakeholders suggested SCC needed to focus on rebranding and involve more diverse stakeholders including grassroots organisations (paradox with perception that professional networks were missing), younger generations and people situated outside the field of health inequalities (paradox with long standing experts feeling devalued and potentially disrespected). The longevity and experience of stakeholders in existing coalitions (Context) may be a barrier to SCC’s progress in the future (Mechanism), and work with a new generation of stakeholders (Outcome).

*Might need to start afresh… recreate some of these coalitions…bring in a new generation of people. So rather than try and kind of like get people who are very established and have done this work for that kind of 50s, in terms of some of the other people who are doing this work to bring in new people who are much younger. You’re coming at it from a different angle. (CEO, Community organisation)*

### From a Theory of Change (TOC) to create an Overarching Programme Theory (OPT)

**Fig 5:**
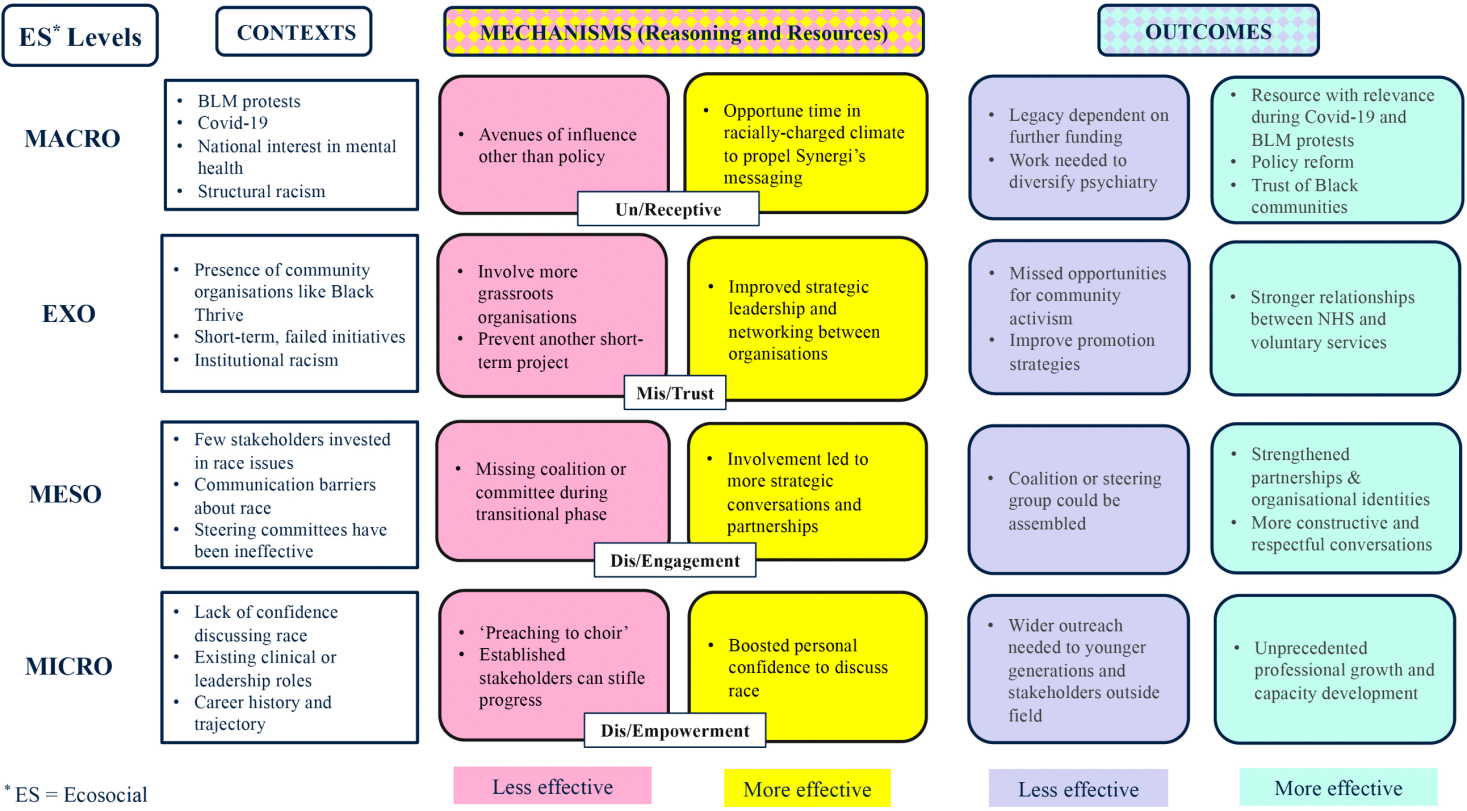
Iterated Programme Theory.

The initial programme theory based on the programmes initial TOC is set out Appendix A in S1 Text. The revised *overarching programme theory* (Fig 5) summarises the CMO configurations and contrasting positions from the analysis. A key contribution of this iteration to develop an OPT was the understanding that continued structural racism and the history of ineffective anti-racism initiatives (Context) raised scepticism about SCC’s legacy and influence (Mechanism), despite recognition that it forged partnerships as a pioneer before and during the pandemic, a time of unprecedented interest in health inequalities. These potentially undermining generative mechanisms of negative outcomes must be managed in future programmes.

Improving SCC’s visibility and outreach was proposed as having a potentially helpful impact on policy and practice (Outcome), alongside engaging new community partners, new albeit less experienced, and younger actors with different types of knowledge. Ensuring all participants had interdisciplinary and co-design competencies, including managing power and knowledge was seen as important for the success of future programmes. In addition temporal factors were likely to impact on the activation of mechanisms impacting outcomes - these related to the duration and history of stakeholder careers, processes for building relational trust between stakeholders, and the unprecedented timing of increased interest in health inequalities coinciding with COVID-19 pandemic after SCC’s launch.

## Discussion

Our approach identified helpful CMO configurations, which might be further tested and scaled in future interventions and evaluations. This is especially important for so called ‘wicked problems’ which are difficult to shift, and interventions that are difficult to evaluate using conventional designs [24,41]. The evaluation also captures a ‘dark logic’ model of CMO configurations revealing what might not go well [42,43]. This knowledge is valuable for the development and implementation of future programmes.

The main findings suggest programme leaders must manage pessimism due to previous programme failures and closures, which eroded trust and confidence; ensure sustainable investment in future programmes and manage the expectations around sustainability; bring in new as well as experienced and well-established leadership and participants whilst managing power, co-design, and potential for disengagement. These findings align with well-known barriers identified in established implementation frameworks [27], although these often lack a focus on ethnic inequalities [44].

As the programme ended, more events and actions followed. We set these out the activities and later impacts in Table 2. The additional impacts suggest that assessment of impact should continue for many years after the completion of a programme. For example, at the closure of SCC, SCC negotiated further funding for a more community lead version of SCC (Synergi 2.0); further research grants were secured to investigate ethnicity and involuntary detention. Monitoring impacts such as this might address some of the concerns about short term funding and less community involvement..

**Table 2:** Logic Model showing intended theory of change and emergent processes and outcomes.

Intended				Actual	
Context	Inputs	Processes and actions	Outputs	Outcomes	Impacts
Decades of ethnic inequalities in experience and outcomes of severe mental illness. Historical programmes have not made headway and left silence around race equality. Black Lives matter Covid-19 Pandemic 2–3 years into the programme	Partnerships with charities. Lived experience experts. Programme management board. Project management team. Communications expertise and strategy. Financial resources.	Creative arts and experiential representation of the disparities. Creative spaces as opportunities to share, explore, learn, build trust and propose solutions. Collaborative Leadership to support systems leaders in specific sites: London, Leeds, Manchester, Birmingham. Briefing papers to clarify terminology and set out the positioning of Synergi around common dilemmas and debates. Working paper on cultural adaptation of psychological therapies interventions. Research including systematic reviews and surveys. Grant applications Commentaries on BLM and Mental Health Bill consultations	Photovoice workshops. Connection between sectors and board room to front line clinicians in four cities in England. Local Synergi networks, the most advanced and self-sustaining one being in Leeds. Leaders from each of the cities emerging and leading local activities in their local health and social care systems. Briefing papers. Publications on syndemics of psychosis, COVID-19 and syndemics, multiple disadvantages, MHA section and ethnicity. NIHR PRP funded research on MHA and ethnicity to inform Mental Health ACT reform. Pieces in lay press and blogs for the public and to promote awareness on ethnic disparities.	A methodology for future experience-based research and funding applications. Further collaborations with arts and communications groups and learning on managing large multi-sector programmes. All cities formed into learning networks, and Synergi Leeds still promoting effective cross sector work. Site specific pledges to implement and review progress. Briefing papers on categories of ethnicity and illness; racism and mental health; priorities for action. Leaders remain active and involved in debate, irrespective of degree of progress; advocating for more inclusive practice and better methods. Contributing to the wider policy and political discourse and setting out conceptual clarifications and position to enable progress across and coalitions across diverse groups. Informed RHO commission to pursue this line of research, with another team undertaking a more detailed analysis. Provide a more complex model of how ethnic disparities arise, and preventive systems and clinical interventions required. Successfully completed research presented to policy makers and informed scrutiny process through the two houses. Additional applications on multimorbidity and ethnicity also on post-partum work, and much on lived experience research. Also informed ATTUNE methods for study of adolescent mental health and adverse childhood experiences.	Capacity building early and mid-career researchers. Establishing new methodologies for EBCD using photovoice and creative arts methods. Readiness for new projects, some from us, some from other groups, on ethnicity and mental health care, and health and social care generally. Charity sector lead a legacy proposal and lead Synergi 2.0 Foregrounding process by which race equality intentions have been removed from the new Bill over time.

### Changing contexts

Since SCC began its work, the Race and Health Observatory was launched, funded directly from the NHS to promote race equality [45]. NHS England launched the patient and carer race equality framework (PCREF) mandating the regional commissioning and strategic organisations to reduce ethnic inequalities, collect data, implement improvement, and monitor progress [46]. The National Institute of Health Research also set out its research inclusion strategy (2022–2027) to improve representation in research. Organisational and policy strategies now prioritise race equality alongside research inclusion and use of appropriate outcomes for protected characteristics under the Equality Act (2010), including influences such as socioeconomic status, geographic location, and access to health and care. Another influential policy directive (CORE20 + 5) for care and research is to address the health of those living in the 20% most deprived areas, and those with five conditions: maternity, severe mental illness, respiratory disease, early cancer diagnosis, and hypertension; a focus on smoking cessation was intended to impact on all five conditions [47].

The NHS plan and the *three shifts* in the UK (analogue to digital, hospital to community, treatment to prevention) risk overlooking the entrenched ethnic inequalities in experiences and outcomes of severe mental illness, as well as intersectional, social, commercial, and political drivers of poor health [48]. Translational and implementation frameworks generally lack considerations of cultural adaptation and ethnic inequalities. These need optimisation to avoid widening inequalities [44].

### Strengths and limitations

This methodology was experimental in nature – to try and make most efficient use of existing data. Post-hoc analyses have a series of weaknesses because the data used was not collected for the purpose of the research. However, this research has shown that programme logic can be surfaced from realist-informed secondary analysis that can be of use in informing future research and or community level activity. Realist-informed secondary analysis is a particularly useful and feasible design for assessing complex systems and programmes, where there are polarising views as it allows for multiple realities (individual) to co-exist and be in tension with one another within a wider reality (system). This prevents superficial analysis of complex programmes of work to surface generative mechanisms that change stakeholder behaviour and lead to or obstruct change. Secondary analyses are becoming increasingly important as a way of maximising the value of existing data, especially as archival storage of qualitative datasets is becoming more common. Secondary analysis is less burdensome, especially for highly sensitive topics.

The novel approach we used does not have agreed reporting standards, although the current study demonstrates the feasibility. The lead analyst (PH) did not participate in data collection, nor in SCC, so the data collection and analysis were not influenced by the original team and reflexively considered their own positionality (social and cultural and professional) whilst interpreting the data. All authors were careful to discuss potential biases in the interpretation of the findings taking account of their experiences, sex, age, ethnicity, and role in the research and SCC. The primary limitation of secondary realist analysis is that we did not evaluate effectiveness of the programme using a classical realist or evaluative design. Instead, we retrospectively explored what elements were *felt* to have been useful or not, and importantly, *why*, in terms of CMO configurations.

## Conclusions

SCC built a strong foundation of knowledge and lasting partnerships and legacy organisations, which continue to contribute to national systems change. We identified important CMO configurations and a programme theory, including dark logic models, that can inform the design and evaluation of future programmes.

## Supporting information

S1 Text Appendix A: Theory of change of SCC. Appendix B: Stakeholder profile. Appendix C: CMO development of advisory boad meetings and annual funder reports. (DOCX)
