# Supplementary material for: Reducing ethnic inequalities in experiences and outcomes of mental illness: A realist informed national programme evaluation
Source: PLOS Ment Health. 2026 Jun 29;3(6):e0000643. doi: 10.1371/journal.pmen.0000643 (PMC13313339; doi:10.1371/journal.pmen.0000643)
Supplement: S1 Text — (DOCX) [file pmen.0000643.s001.docx]

# Appendix A: Theory of Change

# Appendix B: Stakeholder profiles

Interviews were conducted with a total of 24 stakeholders, predominantly within leadership/management (*n* = 21) roles. The following table depicts the professional roles occupied by stakeholders (Row 1) and the institution in which they operated (Column 1). There were several stakeholders (*n* = 5) who held more than one position and were therefore categorised across more than one institution.

**Frequency of stakeholders in each professional role and institution**

|  | **Leadership/**  **Management (n=21)** | **Academic/**  **Research (n=8)** | **Practitioner (n=4)** | **Consultant (n=5)** | **Police service (n=1)** |
| --- | --- | --- | --- | --- | --- |
| **Academia** | 1 | 5 | 0 | 0 | 0 |
| **Government** | 3 | 0 | 0 | 0 | 0 |
| **NHS** | 6 | 1 | 2 | 1 | 0 |
| **Community organisation** | 8 | 2 | 0 | 0 | 0 |
| **Representative groups** | 3 | 0 | 0 | 0 | 0 |
| **Police services** | 0 | 0 | 0 | 0 | 1 |
| **Private/**  **Freelance** | 0 | 0 | 2 | 4 | 0 |

Given that most of the stakeholders interviewed in the stakeholder insight for Synergi held leadership/management roles, this explained the prevalence of their voices across all themes identified from the qualitative analysis.

In the following figure, it is possible to observe the frequency of stakeholders (column) within each institution (row) who provided supporting excerpts for each of the four themes in their interviews.

**Representation of stakeholders and the institutions to which they belonged across each theme**

The figure shows that most of the evidence for Theme 1 (Creation of learning hub) came from stakeholders in community organisations. Most of the evidence for Theme 2 (Growth from new partnerships and collaborations) was from stakeholders in NHS or community organisations, but was consistently endorsed across all groups. Most of the evidence for Theme 3 (Need more attention to legacy) came from stakeholders in NHS or community organisations. Most of the evidence for Theme 4 (Require wider outreach and impact) was provided by stakeholders in academia or community organisations.

# Appendix C: CMO development of Advisory Boad Meetings and Annual Funder Reports

| **Numbered report (by year)** | **Context** | **Mechanism (Resources + Reasoning)** | | **Outcome** |
| --- | --- | --- | --- | --- |
| Year 1 (2017-18) | - Existing loss of trust in authorities - Limited health literacy and understanding of ethnic inequalities - Fatigue from repeated efforts to tackle ethnic inequality - Organisations already face financial constraints and limited partnerships | - Evidence reviews - Collaborations with service user groups, NGOs, statutory services - Funding for public engagement | - Developing understanding of views and expectations - Reconciling or holding tensions or contradictory positions - Increasing commitment to shared agenda - Mobilising change and collaborative leadership - Finding models of relational networks and connecting organisations | - Established trust and partnerships - Built knowledge - New conversations and systems thinking - Some organisations perceived Synergi as a funding body, or as the legacy or an extension of their work - Influenced discussions around the new Mental Health Act review |
| Year 2 (2018-2019) | - Pressures on project team with limited capacity - Challenge engaging advisory board and influencing at national level - Difficulty balancing power dynamics between stakeholders | - Communications Strategy - Recruitment of full project team - PAR - Creative Spaces events - ‘Task and Finish’ group - Impact evaluation | - Bringing lived experiences to forefront - Continuing to push agenda in mental health locally | - Recognition of agentic potential of participation - Broadened strength and use of knowledge amongst collaborative networks - Supported possibility of various stakeholders being brought together to co-produce solutions to challenges in creative spaces |
| Year 3 (2019-2020) | - Power dynamics between stakeholders - Difficulty influencing policy makers | - PAR - Creative Spaces events | - Challenging existing ways of thinking about issues - Increasing knowledge exchange and sharing learning experiences - Co-creating evidence to better understand or document how the issue manifests in different localities, to inform effective intervention | - Academic outputs and poster prize - Continued benefits from collaborative networks |
| Year 4 (2020-2021) | - Global pandemic announced – amplified mental health issues - Team have worked from home, no in-person meetings, staff shortages - Pressures on the team in terms of university and NHS demands, expectations and restrictions - Changing political climate - Murder of George Floyd and consequent Black Lives Matter movement - Research evaluation delayed as ethical committees were not meeting or prioritising non-COVID research | - *Synergi Stories eJournal* launched - captured the experiences of people from ethnic minority backgrounds who have a mental health diagnosis and those who care for them - Launched the UK’s first national pledge - Active media presence - Partnerships with NGOs - ‘Task and Finish’ group - *Synergi* steering group | - Calling on senior leaders in NHS mental health trusts, public bodies and commissioning to declare their commitment to reduce ethnic inequalities in mental health care systems - Finding agreement surrounding the language, messaging and discussions around the legacy of the work - Highlighting the impact of health inequalities | - Important *Synergi* legacy is the NIHR policy research grant on the Mental Health Act - ran over two years from Feb 2021 - Positive feedback from partners |
| Year 5 (2022) | - Broader conversation on structural disadvantage, ethnic inequality and mental health has changed in the last 5 years, but particularly in the last 2 years as a result of the COVID 19 pandemic and the Black Lives Matter movement | - Tangible outputs hosted on website - Realist evaluation of *Synergi* | - Sharing knowledge through creative methods - Influencing and motivating systems change | - Systems work has impacted in specific localities (creative spaces and the pledge) but also modelled for other venues how to progress with the right leadership and approach - Provided a foundation upon which an understanding of how to facilitate local and national systems change can be achieved in the area of ethnic inequalities and mental health - Systems literacy around ethnic inequalities and racism is now much greater |
