# Supplementary material for: Reducing ethnic inequalities in experiences and outcomes of mental illness: A realist informed national programme evaluation
Source: PLOS Ment Health. 2026 Jun 29;3(6):e0000643. doi: 10.1371/journal.pmen.0000643 (PMC13313339; doi:10.1371/journal.pmen.0000643)
Supplement: Attachment — Submitted filename: kb V19 R2R 110326 .docx [file pmen.0000643.s003.docx]

Response to editorial and reviewer comments

| Editorial Comments   1. Please provide additional details regarding participant consent. In the ethics statement in the Methods and online submission information, please ensure that you have specified (1) whether consent was informed and (2) what type you obtained (for instance, written or verbal, and if verbal, how it was documented and witnessed). 2. If your study included minors, state whether you obtained consent from parents or guardians. If the need for consent was waived by the ethics committee, please include this information. 3. If you are reporting a retrospective study of medical records or archived samples, please ensure that you have discussed whether all data were fully anonymized before you accessed them and/or whether the IRB or ethics committee waived the requirement for informed consent. If patients provided informed written consent to have data from their medical records used in research, please include this information. 4. Please amend your detailed Financial Disclosure statement. This is published with the article. It must therefore be completed in full sentences and contain the exact wording you wish to be published. i. Please clarify all sources of financial support for your study. ii. Disclosure.  ii. State the initials, alongside each funding source, of each author to receive each grant. iii. State what role the funders took in the study. If the funders had no role in your study, please state: “The funders had no role in study design, data collection and analysis, decision to publish, or preparation of the manuscript.” iv. If any authors received a salary from any of your funders, please state which authors and which funders.   3. Please provide separate **figure files in .tif or .eps** format.  For more information about figure files please see our guidelines:  [https://journals.plos.org/globalpublichealth/s/figures](https://urldefense.com/v3/__https:/journals.plos.org/globalpublichealth/s/figures__;!!CF15FET90Tp8!Fls26MrtaxB024JFM0S3cJNaue2bfYP8OOwdhqT4X8jdsxkWgR9HSvioqKq6BKvPaAHKn1Mb7mMO70b2VWEA78OEuo0H$)  [https://journals.plos.org/globalpublichealth/s/figures#loc-file-requirements](https://urldefense.com/v3/__https:/journals.plos.org/globalpublichealth/s/figures*loc-file-requirements__;Iw!!CF15FET90Tp8!Fls26MrtaxB024JFM0S3cJNaue2bfYP8OOwdhqT4X8jdsxkWgR9HSvioqKq6BKvPaAHKn1Mb7mMO70b2VWEA7_DBdE1D$)  4. Tables should not be uploaded as individual files. Please remove these files and include the **Tables in your manuscript file as editable, cell-based objects.** For more information about how to format tables, see our guidelines:  [https://journals.plos.org/globalpublichealth/s/tables](https://urldefense.com/v3/__https:/journals.plos.org/globalpublichealth/s/tables__;!!CF15FET90Tp8!Fls26MrtaxB024JFM0S3cJNaue2bfYP8OOwdhqT4X8jdsxkWgR9HSvioqKq6BKvPaAHKn1Mb7mMO70b2VWEA719VE9sH$)  5. We have noticed that you have uploaded Supporting Information files, but you have not included **a list of legends**. Please add a full list of legends for your Supporting Information files after the references list.  6. We note that you have indicated that there are restrictions to data sharing for this study. For studies involving human research participant data or other sensitive data, we encourage authors to share de-identified or anonymized data. However, when data cannot be publicly shared for ethical reasons, we allow authors to make their data sets available upon request. For information on unacceptable data access restrictions, please see [http://journals.plos.org/plosone/s/data-availability#loc-unacceptable-data-access-restrictions](https://urldefense.com/v3/__http:/journals.plos.org/plosone/s/data-availability*loc-unacceptable-data-access-restrictions__;Iw!!CF15FET90Tp8!Fls26MrtaxB024JFM0S3cJNaue2bfYP8OOwdhqT4X8jdsxkWgR9HSvioqKq6BKvPaAHKn1Mb7mMO70b2VWEA74Hjtehy$).     Before we proceed with your manuscript, please address the following prompts:    a) If there are ethical or legal restrictions on sharing a de-identified data set, please explain them in detail (e.g., data contain potentially identifying or sensitive patient information, data are owned by a third-party organization, etc.) and who has imposed them (e.g., a Research Ethics Committee or Institutional Review Board, etc.). Please also provide contact information for a data access committee, ethics committee, or other institutional body to which data requests may be sent.    b) If there are no restrictions, please upload the minimal anonymized data set necessary to replicate your study findings to a stable, public repository and provide us with the relevant URLs, DOIs, or accession numbers. Please see [http://www.bmj.com/content/340/bmj.c181.long](https://urldefense.com/v3/__http:/www.bmj.com/content/340/bmj.c181.long__;!!CF15FET90Tp8!Fls26MrtaxB024JFM0S3cJNaue2bfYP8OOwdhqT4X8jdsxkWgR9HSvioqKq6BKvPaAHKn1Mb7mMO70b2VWEA7-HDhRie$) for guidelines on how to de-identify and prepare clinical data for publication. For a list of recommended repositories, please see [https://journals.plos.org/plosone/s/recommended-repositories](https://urldefense.com/v3/__https:/journals.plos.org/plosone/s/recommended-repositories__;!!CF15FET90Tp8!Fls26MrtaxB024JFM0S3cJNaue2bfYP8OOwdhqT4X8jdsxkWgR9HSvioqKq6BKvPaAHKn1Mb7mMO70b2VWEA77wshu8L$). You also have the option of uploading the data as Supporting Information files, but we would recommend depositing data directly to a data repository if possible.    Please update your Data Availability statement in the submission form accordingly.  Reviewers' comments:  Reviewer's Responses to Questions  **Comments to the Author**  Reviewer #1: Overall Assessment  The manuscript presently faces issues of conceptual and methodological vagueness, framework saturation, and sporadic over-analysis of results. The theoretical framework, analytical methods, and assertions regarding program effectiveness require better clarification before the conclusions can be entirely substantiated.    Major Issues  1. Positioning and clarity of the theoretical framework  The manuscript introduces multiple frameworks: realist evaluation, critical realism, COM-B, socio-ecological model (SEM), and programme theory iteration. While each is individually defensible, their relationships are not clearly specified.  Currently, it is unclear: • whether the study is a realist evaluation, a critical realist qualitative study, or a thematically analysed qualitative evaluation interpreted through realist language; • how COM-B functions analytically (programme theory vs analytic model); • whether SEM is explanatory or merely organisational.  Suggested improvement The authors should explicitly clarify: 1. the ontological and methodological stance (realist evaluation vs critical realism); 2. the analytic role of CMO configurations (primary explanatory mechanism); 3. the role of SEM (organising structure, not explanatory mechanism); 4. the role of COM-B (initial programme theory, not analytic framework).  A short clarifying paragraph in the Introduction (signposting the realist approach) and a tighter, more operational description in Methods would substantially improve coherence.    2. Secondary realist analysis requires stronger justification  The study applies a realist-informed analysis to data originally collected for an independent evaluation. This is innovative but raises validity concerns. The current rationale emphasises efficiency but does not sufficiently address methodological limitations.  Key concerns: • interview guides were not designed to elicit mechanisms; • mechanisms may therefore be inferred rather than observed; • retrospective interpretation risks analytic over-reach.  Suggested improvement The authors should: • acknowledge the limits of identifying mechanisms from non-realist primary data; • explain how they distinguished between participant perceptions and inferred mechanisms; • describe safeguards (e.g., analytic triangulation, negative case analysis, team discussions). Without this, readers cannot assess whether the CMOs are empirically grounded or interpretive constructions.    3. Distinction between perceived outcomes and actual programme impact  Throughout the Results and Discussion, stakeholder perceptions are sometimes presented as evidence of system change. However, the data consist primarily of interviews and meeting minutes rather than service indicators, patient outcomes, or policy implementation evidence.  For example, claims regarding “system reform,” “national systems change,” or “impact on policy and practice” appear stronger than the data support.  Suggested improvement The authors should consistently distinguish: • perceived influence, • organisational engagement, • and demonstrable system change. Rephrasing conclusions as “**perceived contributions to system change**” or “mechanisms likely to enable change” would align claims with evidence.  4. Operationalisation of Context, Mechanism, and Outcome  The paper defines CMO categories but does not consistently apply them. In several instances: • slow change is described as a mechanism, • historical context is treated as an outcome, • or programme activities are labelled mechanisms.  **Mechanisms in realist evaluation should be generative processes** (e.g., trust, scepticism, disengagement, empowerment) rather than events or conditions.  Suggested improvement Provide a clear coding rule (with examples) distinguishing: • Context = conditions and structures • Mechanism = reasoning + responses triggered by the programme • Outcome = resulting changes in behaviour, relationships, or practice This clarification is essential for methodological credibility.  5. Framework overload and analytic redundancy  The manuscript simultaneously uses: • thematic analysis, • CMO analysis, • SEM layering, • and “three ears of listening.” This risks appearing methodologically unfocused and may weaken confidence in analytic rigour.  Suggested improvement Clarify hierarchy: • CMO reasoning = primary analytic strategy • thematic analysis = coding process • SEM = presentation structure The “three ears of listening” requires stronger justification or simplification, as it currently appears interpretively subjective.  6. Sampling and selection bias  Of 44 original interviewees, only 24/25 consented to secondary analysis. The manuscript does not assess whether this group differs systematically from those who declined. This is important because engaged stakeholders are more likely to respond positively to programmes.  Suggested improvement Provide: • role distribution of participants, • representation across sectors, • and a short statement about potential selection bias.    7. Discussion and policy implications  The Discussion provides thoughtful interpretation but **sometimes shifts into advocacy.** Recommendations would be stronger if directly mapped to specific mechanisms identified in the findings.  Suggested improvement Structure recommendations using CMO logic, e.g.: • Context: historical programme failure → Mechanism: pessimism → Recommendation: long-term funding commitments and legacy planning • Context: expert–lived experience tension → Mechanism: disengagement → Recommendation: structured co-design governance    Minor Issues 1. Inconsistent participant numbers (24 vs 25) should be corrected throughout. 2. Interview dates (2020 vs 2021) require clarification. 3. Some typographical errors (e.g., duplicated words, minor grammatical issues).  4. The Introduction could be shortened; historical policy review is somewhat long relative to study aims.  5. Provide more detail about programme outputs and activities (what SCC actually did operationally).  6. Clarify the function of advisory board minutes (corroboration vs contextual background).  7. Strengthen reflexivity statement given that two authors were involved in programme delivery.  8. Avoid overuse of specialised terminology where plain language would improve accessibility.  9. Figures (ToC and CMO diagrams) should be interpretable without referring extensively to appendices.  10. Some policy frameworks mentioned later in the paper should be supported with citations.  Figure Resubmissions:  While revising your submission, we strongly recommend that you use PLOS’s NAAS tool ([https://ngplosjournals.page](https://urldefense.com/v3/__https:/ngplosjournals.pagemajik.ai/artanalysis__;!!CF15FET90Tp8!Fls26MrtaxB024JFM0S3cJNaue2bfYP8OOwdhqT4X8jdsxkWgR9HSvioqKq6BKvPaAHKn1Mb7mMO70b2VWEA7zlNTUSf$)  [majik.ai/artanalysis](https://urldefense.com/v3/__https:/ngplosjournals.pagemajik.ai/artanalysis__;!!CF15FET90Tp8!Fls26MrtaxB024JFM0S3cJNaue2bfYP8OOwdhqT4X8jdsxkWgR9HSvioqKq6BKvPaAHKn1Mb7mMO70b2VWEA7zlNTUSf$)) to test your figure files. NAAS can convert your figure files to the TIFF file type and meet basic requirements (such as print size, resolution), or provide you with a report on issues that do not meet our requirements and that NAAS cannot fix.  After uploading your figures to PLOS’s NAAS tool - [https://ngplosjournals.pagemajik.ai/artanalysis](https://urldefense.com/v3/__https:/ngplosjournals.pagemajik.ai/artanalysis__;!!CF15FET90Tp8!Fls26MrtaxB024JFM0S3cJNaue2bfYP8OOwdhqT4X8jdsxkWgR9HSvioqKq6BKvPaAHKn1Mb7mMO70b2VWEA7zlNTUSf$), NAAS will process the files provided and display the results in the "Uploaded Files" section of the page as the processing is complete. If the uploaded figures meet our requirements (or NAAS is able to fix the files to meet our requirements), the figure will be marked as "fixed" above. If NAAS is unable to fix the files, a red "failed" label will appear above. When NAAS has confirmed that the figure files meet our requirements, please download the file via the download option, and include these NAAS processed figure files when submitting your revised manuscript. | Thank you. We have set out in greater detail what the consent process was in the methods. This was at the time of COVID, hence there was a delay in processing of ethics approval. We note the consent was by email, confirming the transcript could be entered into research for an interview that was not a research interview but was part of an independent evaluation. Ethics approval for the secondary analysis was issued in 2023, even though data collection was complete in 2020.  The study did not include minors.  We received transcripts and stakeholder roles rather than a full set of demographic details. IRB that approved of the study were aware of these constraints. We did not use medical records.  We have amended the conflicts of interest statement in which we make financial disclosures, and expanded it to include the funding source, who was funded, and also later sources of funding which were not directly linked with but emerged from SCC.  We have amended the figure file to a TIF file.  The tables have been added into the main paper and are editable. These include text rather than numerals, given the nature of the study.  We now provide a legend in the main paper, listing the appendix files (supplementary information).  The original data collection was a qualitative evaluation, including situational data. The secondary analysis we undertook was supported by the ethics committee, however, consent did not extend to releasing the transcripts beyond the research process. There were several constraints at the time of the study evaluation due to the COVID-19 pandemic, including delays in ethics processing and in person and new interviews were not encouraged. Hence, we relied on a secondary analysis. We are not permitted, by the original permission to release data for research secured by the evaluation team (Mel Larson and associates). These are ethical restrictions, but legal frameworks also guide reasonable use of data. Given the qualitative nature of the data it may be possible for a third party to identify individual from the location and types of information and the role, and the timing of the study. Therefore, in our secondary analysis we report aggregated descriptive data. Our data availability statement addresses these points.  We have added data availability to the manuscript file and will also add this to the online system.  Thank you for reviewing this paper, and for all the positive comments. Here we focus on the suggestions for improvement and respond to them, indicating how we’ve altered the text.  Thank you. We have reviewed the entire manuscript and have simplified the language and clarified the methodological ontological underpinnings and linked theoretical frameworks used to inform analysis of the data (see theoretical approach p 8 of tracked ms). We also introduce Figure 2, which sets out which frameworks were used and for which purpose during the programme itself, the external evaluation, and this our realist-informed secondary analysis. We appreciate this method is complex as it is post-hoc evaluation and we have therefore clarified the analytic stance and methods and reviewed the data from the previous figures 2 and 3 (now figures 3 and 4) and edited the paper to focus on the strongest findings.  Thank you. Figure 2 and the text now make clearer that the COM-B model was part of the original theory of change for the programme as it was developed and delivered, rather than it being central to the emergent findings. We have clarified this in aims and objectives and in Figure 2.  This was a realist informed secondary analysis, making use of the related SEM levels as an organising and explanatory framework of relevance for the emergent findings. See page 10 of tracked ms.  1,2.3: We have set out the ontological and methodological stance now, and the role of CMOs. We explain the way SEM is applied in the analysis and why (see page 8/9 of tracked ms).  4.As outlined above, we emphasise the COM-B model was part of original theory of change but not relevant to the analysis or findings, which took a systems rather than individual behavioural perspective.  Thankyou, we have done this mainly in the methods section, and in the analysis section as the introduction is setting the context and history of tackling inequalities. We do make reference to realist informed secondary analysis/evaluation early on. We have enhanced the limitations section to reflect these concerns (see pages 8/9, 12 of tracked ms.  We agree, and draw out the limitations of the approach, which we also say is also potentially interesting methodologically. We explain why the approach was adopted. This was partly through necessity around the period of the COVID pandemic when ethics committees were not prioritising anything but COVID research. We were not able to follow new data collection plans, rather we made use of data already collected. The approach was ground in previous methodological advances from team members, which are now referenced. (see page 10 of tracked ms)  Generative mechanisms by their nature are inferred rather than directly observed, and we have made this clearer in the analysis and discussion. We draw out the main findings of importance based on stakeholder testimony through their interviews, and we seek to use their own understanding as a window into the programme. In layering together their understandings and narratives we have been able to surface their logic for behaviour change or inertia and draw on their reasoning to inform why that has taken place. This has helped generate learning and understanding about how to deliver such programmes in the future. We accept the data are based on perceptions, yet the inferred mechanisms emerged in the data; the methods for doing this are now set out clearly (see p 12, 13 of tracked ms).  We have added a section on team discussion, although the analysis was undertaken independently of the authors who were involved in Synergi (in methods and on p25 of tracked ms).  Thank you. Yes we agree, this is an important distinction. We have reviewed the language and moderated to try and be clearer – the CMO configurations were developed consistently as a core part of the secondary analysis and it is these that helped to inform our overarching programme theory. We are aware that we cannot assess what *real* impacts on patient care were witnessed, other than through the eyes of stakeholders. However, the evaluation and our paper is largely focused on the programme and perceptions of where the programme itself worked well and when not at the programme level. We have modified the conclusions and discussion to reflect this.  We thank you for this suggestion about how to improve clarity of methods (see p. 13 of tracked ms) We have rewritten the methodology to be clearer about how the methodological tools (CMO analysis including three ears of listening) were used informed by theory (SEM).  We have reviewed all CMO categories and modified where there were ambiguities, or lack of clarity; likewise we have reviewed all identified mechanisms and labelled them.  We define these three clearly and set out how coded (p 13).  Thanks, as outlined above, we have added Figure 2, and been more careful about how the different frameworks were used. See methods section.  We have explained we are unable to determine why the others declined to take part – however – the shadow theory emerged as we were able to use transcripts from participants with both positive and negative framing of mechanisms and outcomes. Whilst no research can rule out selection bias and we have amended the limitations to acknowledge this; we cannot be sure in this instance that this is bias rather than attrition as is common research. Given the limitations around ethical approval to access data only from those consenting, we can not secure further information from those who declined to have their transcript passed to us originally, and then those who later declined to consent when we contacted them.  We did provide role and sector distribution in an appendix for the participants.  Thank you. As outlined, we have simplified and explained the various frameworks and their use. We reference the three ears of listening methodology used in a previous programme.  Thank you. We have reviewed the discussion with care and minimised drift into general advocacy, other than raising the concerns about how complex such programmes are and how rarely they are evaluated. We also emphasise the implications of inaction. The inequalities are persistent, and hence we do emphasise we need to continue to find ways of tackling these. We have indeed set out with care the CMO logic referred to in narrative form in the results, drawing on the important ones in the discussion (see also a revised Figure 5). We could add more figures/schematics if helpful, but we already have 5 figures and felt more might be excessive.  Thank you. Apologies for the inconsistencies and typos. These have been corrected.  We have corrected the errors. We analysed 24 transcripts of 27 who gave permission for their transcript to be shared but not all consented to actual use when we approached them. Regrettably we do not have information on the full 44 or the 27, only the 24 that agreed. We have included comment of this in the limitations section.  The introduction: we have shortened this, yet we also felt it important to set out what had been tried previously and the lessons. That was the historical context in which the SCC was commissioned.  We have provided, in Table 2, more information on the outputs over a longer time period. Our revised theory of change in Figure 5 also sets this out.  We have clarified the role of the AB and how the minutes and documents were used to corroborate and cross check findings from the transcripts.  We have added a reflexivity section in the limitations.  We have tried to reduce any jargon.  The figures have been revised and submitted as tif files.  We have added some citations to support our account of what developed after SCC. We did not reference government policies as these are easily found on the internet, and this would have added more references. We have expanded the references to reflect the greater detail now provided, and to strengthen the methodological grounding.  Thank you. We have revised and submitted figures as tif files and generated the figures using the tool as adjusted to suit requirements. |
| --- | --- |
