# Supplementary material for: Reducing ethnic inequalities in experiences and outcomes of mental illness: A realist informed national programme evaluation
Source: PLOS Ment Health. 2026 Jun 29;3(6):e0000643. doi: 10.1371/journal.pmen.0000643 (PMC13313339; doi:10.1371/journal.pmen.0000643)
Supplement: Attachment — Submitted filename: We are delighted the reviewer found the paper of interest.docx [file pmen.0000643.s004.docx]

We are delighted the reviewer found the paper of interest. Here is our point by point response.

There two minor comments the reviewer raises and we respond to these points here.

1) Comment: Page 14, the quote did not make sense. Our response: It is usual to not edit quotes from participants in qualitative research to avoid risk of misinterpreting the quote. In this instance, we agree the quote is verbatim and although adhering to this principle, it is not easy to follow. We have edited the quote a little to remove words that appear to be points of reflection or word finding for the participant. The point being made was that the participant found our work provided learning in the form of acquiring a language to talk about the inequalities. We have edited this – visible in the tracked version – to make this clearer, by removing redundant words.
2) Apologies, yes that quote on page 15 should have said ‘get’ not ‘et’. We have amended this now.
