# Supplementary material for: Reducing ethnic inequalities in experiences and outcomes of mental illness: A realist informed national programme evaluation
Source: PLOS Ment Health. 2026 Jun 29;3(6):e0000643. doi: 10.1371/journal.pmen.0000643 (PMC13313339; doi:10.1371/journal.pmen.0000643)
Supplement: Peer review history

# Peer review: Decision Letter 0

**DOI:** 10.1371/journal.pmen.0000643.r001

**Authors:** Steve Zimmerman

19 Feb 2026

PMEN-D-25-00601

Reducing ethnic inequalities in experiences and outcomes of mental illness: a realist informed national programme evaluation.

PLOS Mental Health

Dear Dr. Bhui CBE,

Thank you for submitting your manuscript to PLOS Mental Health. After careful consideration, we feel that it has merit but does not fully meet PLOS Mental Health’s publication criteria as it currently stands. Therefore, we invite you to submit a revised version of the manuscript that addresses the points raised during the review process.

Please note that we have only been able to secure a single reviewer to assess your manuscript. We are issuing a decision on your manuscript at this point to prevent further delays in the evaluation of your manuscript. Please be aware that the editor who handles your revised manuscript might find it necessary to invite additional reviewers to assess this work once the revised manuscript is submitted. However, we will aim to proceed on the basis of this single review if possible.

The reviewer's comments can be found below. Could you please carefully revise the manuscript to address all issues raised?

Please submit your revised manuscript by Apr 04 2026 11:59PM. If you will need more time than this to complete your revisions, please reply to this message or contact the journal office at mentalhealth@plos.org. When you're ready to submit your revision, log on to https://www.editorialmanager.com/pmen/ and select the 'Submissions Needing Revision' folder to locate your manuscript file.

Please include the following items when submitting your revised manuscript:

- A letter that responds to each point raised by the editor and reviewer(s). You should upload this letter as a separate file labeled 'Response to Reviewers'.
- A marked-up copy of your manuscript that highlights changes made to the original version. You should upload this as a separate file labeled 'Revised Manuscript with Track Changes'.
- An unmarked version of your revised paper without tracked changes. You should upload this as a separate file labeled 'Manuscript'.

Guidelines for resubmitting your figure files are available below the reviewer comments at the end of this letter.

We look forward to receiving your revised manuscript.

Kind regards,

Steve Zimmerman, PhD

PLOS Staff Editor

Journal Requirements:

1. Please provide additional details regarding participant consent. In the ethics statement in the Methods and online submission information, please ensure that you have specified (1) whether consent was informed and (2) what type you obtained (for instance, written or verbal, and if verbal, how it was documented and witnessed). If your study included minors, state whether you obtained consent from parents or guardians. If the need for consent was waived by the ethics committee, please include this information.

If you are reporting a retrospective study of medical records or archived samples, please ensure that you have discussed whether all data were fully anonymized before you accessed them and/or whether the IRB or ethics committee waived the requirement for informed consent. If patients provided informed written consent to have data from their medical records used in research, please include this information.

2. Please amend your detailed Financial Disclosure statement. This is published with the article. It must therefore be completed in full sentences and contain the exact wording you wish to be published.

i. Please clarify all sources of financial support for your study. List the grants, grant numbers, and organizations that funded your study, including funding received from your institution. Please note that suppliers of material support, including research materials, should be recognized in the Acknowledgements section rather than in the Financial Disclosure.

ii. State the initials, alongside each funding source, of each author to receive each grant. For example: "This work was supported by the National Institutes of Health (####### to AM; ###### to CJ) and the National Science Foundation (###### to AM)."

iii. State what role the funders took in the study. If the funders had no role in your study, please state: “The funders had no role in study design, data collection and analysis, decision to publish, or preparation of the manuscript.”

iv. If any authors received a salary from any of your funders, please state which authors and which funders.

3. Please provide separate figure files in .tif or .eps format.

For more information about figure files please see our guidelines:

https://journals.plos.org/globalpublichealth/s/figures

https://journals.plos.org/globalpublichealth/s/figures#loc-file-requirements

4. Tables should not be uploaded as individual files. Please remove these files and include the Tables in your manuscript file as editable, cell-based objects. For more information about how to format tables, see our guidelines:

https://journals.plos.org/globalpublichealth/s/tables

5. We have noticed that you have uploaded Supporting Information files, but you have not included a list of legends. Please add a full list of legends for your Supporting Information files after the references list.

6. We note that you have indicated that there are restrictions to data sharing for this study. For studies involving human research participant data or other sensitive data, we encourage authors to share de-identified or anonymized data. However, when data cannot be publicly shared for ethical reasons, we allow authors to make their data sets available upon request. For information on unacceptable data access restrictions, please see http://journals.plos.org/plosone/s/data-availability#loc-unacceptable-data-access-restrictions.

Before we proceed with your manuscript, please address the following prompts:

a) If there are ethical or legal restrictions on sharing a de-identified data set, please explain them in detail (e.g., data contain potentially identifying or sensitive patient information, data are owned by a third-party organization, etc.) and who has imposed them (e.g., a Research Ethics Committee or Institutional Review Board, etc.). Please also provide contact information for a data access committee, ethics committee, or other institutional body to which data requests may be sent.

b) If there are no restrictions, please upload the minimal anonymized data set necessary to replicate your study findings to a stable, public repository and provide us with the relevant URLs, DOIs, or accession numbers. Please see http://www.bmj.com/content/340/bmj.c181.long for guidelines on how to de-identify and prepare clinical data for publication. For a list of recommended repositories, please see https://journals.plos.org/plosone/s/recommended-repositories. You also have the option of uploading the data as Supporting Information files, but we would recommend depositing data directly to a data repository if possible.

Please update your Data Availability statement in the submission form accordingly.

If the reviewer comments include a recommendation to cite specific previously published works, please review and evaluate these publications to determine whether they are relevant and should be cited. There is no requirement to cite these works unless the editor has indicated otherwise.

Additional Editor Comments (if provided):

[Note: HTML markup is below. Please do not edit.]

Reviewers' comments:

Reviewer's Responses to Questions

**Comments to the Author**

1. Does this manuscript meet PLOS Mental Health’s publication criteria? Is the manuscript technically sound, and do the data support the conclusions? The manuscript must describe methodologically and ethically rigorous research with conclusions that are appropriately drawn based on the data presented.

Reviewer #1: Yes

**********

2. Has the statistical analysis been performed appropriately and rigorously?

Reviewer #1: N/A

**********

3. Have the authors made all data underlying the findings in their manuscript fully available (please refer to the Data Availability Statement at the start of the manuscript PDF file)?

The PLOS Data policy requires authors to make all data underlying the findings described in their manuscript fully available without restriction, with rare exception. The data should be provided as part of the manuscript or its supporting information, or deposited to a public repository. For example, in addition to summary statistics, the data points behind means, medians and variance measures should be available. If there are restrictions on publicly sharing data—e.g. participant privacy or use of data from a third party—those must be specified.

Reviewer #1: Yes

**********

4. Is the manuscript presented in an intelligible fashion and written in standard English?

PLOS Mental Health does not copyedit accepted manuscripts, so the language in submitted articles must be clear, correct, and unambiguous. Any typographical or grammatical errors should be corrected at revision, so please note any specific errors here.

Reviewer #1: Yes

**********

5. Review Comments to the Author

Please use the space provided to explain your answers to the questions above. You may also include additional comments for the author, including concerns about dual publication, research ethics, or publication ethics. (Please upload your review as an attachment if it exceeds 20,000 characters)

Reviewer #1: Overall Assessment

This paper offers a qualitative, realist-informed secondary assessment of the Synergi Collaborative Centre (SCC), a national program focused on minimizing ethnic disparities in mental health services. The subject is crucial, current, and relevant to policy, especially in light of persistent worries regarding coercive methods of accessing care among ethnic minority groups. The research is likewise ambitious in its effort to combine realist reasoning, socio-ecological structure, and program theory enhancement within a complicated systems intervention.

The primary advantage of the paper is its attempt to go beyond simple descriptive stakeholder feedback, aiming instead to clarify why programs succeed or fail using Context-Mechanism-Outcome (CMO) reasoning. The analysis at multiple levels and the incorporation of both facilitating and hindering mechanisms significantly enhance research on implementation and health disparities.

Nonetheless, the manuscript presently faces issues of conceptual and methodological vagueness, framework saturation, and sporadic over-analysis of results. The theoretical framework, analytical methods, and assertions regarding program effectiveness require better clarification before the conclusions can be entirely substantiated.

I therefore recommend Major Revision.

Major Issues

1. Positioning and clarity of the theoretical framework

The manuscript introduces multiple frameworks: realist evaluation, critical realism, COM-B, socio-ecological model (SEM), and programme theory iteration. While each is individually defensible, their relationships are not clearly specified.

Currently, it is unclear:

• whether the study is a realist evaluation, a critical realist qualitative study, or a thematically analysed qualitative evaluation interpreted through realist language;

• how COM-B functions analytically (programme theory vs analytic model);

• whether SEM is explanatory or merely organisational.

Suggested improvement

The authors should explicitly clarify:

1. the ontological and methodological stance (realist evaluation vs critical realism);

2. the analytic role of CMO configurations (primary explanatory mechanism);

3. the role of SEM (organising structure, not explanatory mechanism);

4. the role of COM-B (initial programme theory, not analytic framework).

A short clarifying paragraph in the Introduction (signposting the realist approach) and a tighter, more operational description in Methods would substantially improve coherence.

2. Secondary realist analysis requires stronger justification

The study applies a realist-informed analysis to data originally collected for an independent evaluation. This is innovative but raises validity concerns. The current rationale emphasises efficiency but does not sufficiently address methodological limitations.

Key concerns:

• interview guides were not designed to elicit mechanisms;

• mechanisms may therefore be inferred rather than observed;

• retrospective interpretation risks analytic over-reach.

Suggested improvement

The authors should:

• acknowledge the limits of identifying mechanisms from non-realist primary data;

• explain how they distinguished between participant perceptions and inferred mechanisms;

• describe safeguards (e.g., analytic triangulation, negative case analysis, team discussions).

Without this, readers cannot assess whether the CMOs are empirically grounded or interpretive constructions.

3. Distinction between perceived outcomes and actual programme impact

Throughout the Results and Discussion, stakeholder perceptions are sometimes presented as evidence of system change. However, the data consist primarily of interviews and meeting minutes rather than service indicators, patient outcomes, or policy implementation evidence.

For example, claims regarding “system reform,” “national systems change,” or “impact on policy and practice” appear stronger than the data support.

Suggested improvement

The authors should consistently distinguish:

• perceived influence,

• organisational engagement,

• and demonstrable system change.

Rephrasing conclusions as “perceived contributions to system change” or “mechanisms likely to enable change” would align claims with evidence.

4. Operationalisation of Context, Mechanism, and Outcome

The paper defines CMO categories but does not consistently apply them. In several instances:

• slow change is described as a mechanism,

• historical context is treated as an outcome,

• or programme activities are labelled mechanisms.

Mechanisms in realist evaluation should be generative processes (e.g., trust, scepticism, disengagement, empowerment) rather than events or conditions.

Suggested improvement

Provide a clear coding rule (with examples) distinguishing:

• Context = conditions and structures

• Mechanism = reasoning + responses triggered by the programme

• Outcome = resulting changes in behaviour, relationships, or practice

This clarification is essential for methodological credibility.

5. Framework overload and analytic redundancy

The manuscript simultaneously uses:

• thematic analysis,

• CMO analysis,

• SEM layering,

• and “three ears of listening.”

This risks appearing methodologically unfocused and may weaken confidence in analytic rigour.

Suggested improvement

Clarify hierarchy:

• CMO reasoning = primary analytic strategy

• thematic analysis = coding process

• SEM = presentation structure

The “three ears of listening” requires stronger justification or simplification, as it currently appears interpretively subjective.

6. Sampling and selection bias

Of 44 original interviewees, only 24/25 consented to secondary analysis. The manuscript does not assess whether this group differs systematically from those who declined.

This is important because engaged stakeholders are more likely to respond positively to programmes.

Suggested improvement

Provide:

• role distribution of participants,

• representation across sectors,

• and a short statement about potential selection bias.

7. Discussion and policy implications

The Discussion provides thoughtful interpretation but sometimes shifts into advocacy. Recommendations would be stronger if directly mapped to specific mechanisms identified in the findings.

Suggested improvement

Structure recommendations using CMO logic, e.g.:

• Context: historical programme failure → Mechanism: pessimism → Recommendation: long-term funding commitments and legacy planning

• Context: expert–lived experience tension → Mechanism: disengagement → Recommendation: structured co-design governance

Minor Issues

1. Inconsistent participant numbers (24 vs 25) should be corrected throughout.

2. Interview dates (2020 vs 2021) require clarification.

3. Some typographical errors (e.g., duplicated words, minor grammatical issues).

4. The Introduction could be shortened; historical policy review is somewhat long relative to study aims.

5. Provide more detail about programme outputs and activities (what SCC actually did operationally).

6. Clarify the function of advisory board minutes (corroboration vs contextual background).

7. Strengthen reflexivity statement given that two authors were involved in programme delivery.

8. Avoid overuse of specialised terminology where plain language would improve accessibility.

9. Figures (ToC and CMO diagrams) should be interpretable without referring extensively to appendices.

10. Some policy frameworks mentioned later in the paper should be supported with citations.

**********

6. PLOS authors have the option to publish the peer review history of their article (what does this mean?). If published, this will include your full peer review and any attached files.

**Do you want your identity to be public for this peer review?**  If you choose “no”, your identity will remain anonymous but your review may still be made public.

For information about this choice, including consent withdrawal, please see our Privacy Policy.

Reviewer #1: **Yes:**  Israel Fisseha

**********

[NOTE: If reviewer comments were submitted as an attachment file, they will be attached to this email and accessible via the submission site. Please log into your account, locate the manuscript record, and check for the action link "View Attachments". If this link does not appear, there are no attachment files.]

-->Figure Resubmissions:

While revising your submission, we strongly recommend that you use PLOS’s NAAS tool (https://ngplosjournals.pagemajik.ai/artanalysis) to test your figure files. NAAS can convert your figure files to the TIFF file type and meet basic requirements (such as print size, resolution), or provide you with a report on issues that do not meet our requirements and that NAAS cannot fix.

After uploading your figures to PLOS’s NAAS tool - https://ngplosjournals.pagemajik.ai/artanalysis, NAAS will process the files provided and display the results in the "Uploaded Files" section of the page as the processing is complete. If the uploaded figures meet our requirements (or NAAS is able to fix the files to meet our requirements), the figure will be marked as "fixed" above. If NAAS is unable to fix the files, a red "failed" label will appear above. When NAAS has confirmed that the figure files meet our requirements, please download the file via the download option, and include these NAAS processed figure files when submitting your revised manuscript.

# Author comment: Author response to Decision Letter 1

**DOI:** 10.1371/journal.pmen.0000643.r002

24 Mar 2026

Attachment Submitted filename: kb V19 R2R 110326 .docx

# Peer review: Decision Letter 1

**DOI:** 10.1371/journal.pmen.0000643.r003

**Authors:** Steve Zimmerman, Karli Montague-Cardoso

29 May 2026

PMEN-D-25-00601R1

Reducing ethnic inequalities in experiences and outcomes of mental illness: a realist informed national programme evaluation.

PLOS Mental Health

Dear Dr. Bhui CBE,

Thank you for submitting your revised manuscript to PLOS Mental Health and thank you again for your understanding as we tried to secure a second reviewer. I am happy to say that we have now secured a report from the second reviewer and given the stage that the manuscript is at, we would like to give you the opportunity to address the very minor comments raised by the reviewer, which will then be assessed in-house upon resubmission for efficiency.

Please submit your revised manuscript by Jun 27 2026 11:59PM. If you will need more time than this to complete your revisions, please reply to this message or contact the journal office at mentalhealth@plos.org. When you're ready to submit your revision, log on to https://www.editorialmanager.com/pmen/ and select the 'Submissions Needing Revision' folder to locate your manuscript file.

Please include the following items when submitting your revised manuscript:

- A letter that responds to each point raised by the editor and reviewer(s). You should upload this letter as a separate file labeled 'Response to Reviewers'.
- A marked-up copy of your manuscript that highlights changes made to the original version. You should upload this as a separate file labeled 'Revised Manuscript with Track Changes'.
- An unmarked version of your revised paper without tracked changes. You should upload this as a separate file labeled 'Manuscript'.

Guidelines for resubmitting your figure files are available below the reviewer comments at the end of this letter.

As the corresponding author, your ORCID iD is verified in the submission system and will appear in the published article. PLOS supports the use of ORCID, and we encourage all coauthors to register for an ORCID iD and use it as well. Please encourage your coauthors to verify their ORCID iD within the submission system before final acceptance, as unverified ORCID iDs will not appear in the published article. *Only*  the individual author can complete the verification step; PLOS staff *cannot*  verify ORCID iDs on behalf of authors.

We look forward to receiving your revised manuscript.

Kind regards,

Karli Montague-Cardoso

Staff Editor

PLOS Mental Health

**Journal Requirements:**

If the reviewer comments include a recommendation to cite specific previously published works, please review and evaluate these publications to determine whether they are relevant and should be cited. There is no requirement to cite these works unless the editor has indicated otherwise.

Please review your reference list to ensure that it is complete and correct. If you have cited papers that have been retracted, please include the rationale for doing so in the manuscript text, or remove these references and replace them with relevant current references. Any changes to the reference list should be mentioned in the rebuttal letter that accompanies your revised manuscript. If you need to cite a retracted article, indicate the article’s retracted status in the References list and also include a citation and full reference for the retraction notice.

**Additional Editor Comments (if provided):**

[Note: HTML markup is below. Please do not edit.]

Reviewers' comments:

Reviewer's Responses to Questions

**Comments to the Author**

1. If the authors have adequately addressed your comments raised in a previous round of review and you feel that this manuscript is now acceptable for publication, you may indicate that here to bypass the “Comments to the Author” section, enter your conflict of interest statement in the “Confidential to Editor” section, and submit your "Accept" recommendation.

Reviewer #2: (No Response)

**********

2. Does this manuscript meet PLOS Mental Health’s publication criteria? Is the manuscript technically sound, and do the data support the conclusions? The manuscript must describe methodologically and ethically rigorous research with conclusions that are appropriately drawn based on the data presented.

Reviewer #2: Yes

**********

3. Has the statistical analysis been performed appropriately and rigorously?

Reviewer #2: N/A

**********

4. Have the authors made all data underlying the findings in their manuscript fully available (please refer to the Data Availability Statement at the start of the manuscript PDF file)?

The PLOS Data policy requires authors to make all data underlying the findings described in their manuscript fully available without restriction, with rare exception. The data should be provided as part of the manuscript or its supporting information, or deposited to a public repository. For example, in addition to summary statistics, the data points behind means, medians and variance measures should be available. If there are restrictions on publicly sharing data—e.g. participant privacy or use of data from a third party—those must be specified.

Reviewer #2: Yes

**********

5. Is the manuscript presented in an intelligible fashion and written in standard English?

PLOS Mental Health does not copyedit accepted manuscripts, so the language in submitted articles must be clear, correct, and unambiguous. Any typographical or grammatical errors should be corrected at revision, so please note any specific errors here.

Reviewer #2: Yes

**********

6. Review Comments to the Author

Please use the space provided to explain your answers to the questions above. You may also include additional comments for the author, including concerns about dual publication, research ethics, or publication ethics. (Please upload your review as an attachment if it exceeds 20,000 characters)

**Reviewer #2:**  This is a very clear and well written paper. I can see that this is the second revision, so I'm only going to pick up on two very small things. On page 14, I don't understand the first quote at all. Is this misquoted? Or is there some nuance that I'm not seeing?

On page 15 in the quote it says 'et' I think this is supposed to be get?

I really enjoyed reading this paper and found myself nodding along in recognition of the results! I enjoyed the realist aspect as well and could see how this explores the topic and the multiple layers of the issue in a novel way.

**********

7. PLOS authors have the option to publish the peer review history of their article (what does this mean?). If published, this will include your full peer review and any attached files.

**Do you want your identity to be public for this peer review?**  If you choose “no”, your identity will remain anonymous but your review may still be made public.

For information about this choice, including consent withdrawal, please see our Privacy Policy.

Reviewer #2: **Yes:**  Dr Kim Heyes

**********

[NOTE: If reviewer comments were submitted as an attachment file, they will be attached to this email and accessible via the submission site. Please log into your account, locate the manuscript record, and check for the action link "View Attachments". If this link does not appear, there are no attachment files.]

**Figure Resubmissions:**

While revising your submission, we strongly recommend that you use PLOS’s NAAS tool (https://ngplosjournals.pagemajik.ai/artanalysis) to test your figure files. NAAS can convert your figure files to the TIFF file type and meet basic requirements (such as print size, resolution), or provide you with a report on issues that do not meet our requirements and that NAAS cannot fix.

After uploading your figures to PLOS’s NAAS tool - https://ngplosjournals.pagemajik.ai/artanalysis, NAAS will process the files provided and display the results in the "Uploaded Files" section of the page as the processing is complete. If the uploaded figures meet our requirements (or NAAS is able to fix the files to meet our requirements), the figure will be marked as "fixed" above. If NAAS is unable to fix the files, a red "failed" label will appear above. When NAAS has confirmed that the figure files meet our requirements, please download the file via the download option, and include these NAAS processed figure files when submitting your revised manuscript.

# Author comment: Author response to Decision Letter 2

**DOI:** 10.1371/journal.pmen.0000643.r004

1 Jun 2026

Attachment Submitted filename: We are delighted the reviewer found the paper of interest.docx

# Editor report: Decision Letter 2

**DOI:** 10.1371/journal.pmen.0000643.r005

**Authors:** Steve Zimmerman, Karli Montague-Cardoso, Karli Montague-Cardoso

3 Jun 2026

Reducing ethnic inequalities in experiences and outcomes of mental illness: a realist informed national programme evaluation.

PMEN-D-25-00601R2

Dear Professor Bhui CBE,

We are pleased to inform you that your manuscript 'Reducing ethnic inequalities in experiences and outcomes of mental illness: a realist informed national programme evaluation.' has been provisionally accepted for publication in PLOS Mental Health.

Before your manuscript can be formally accepted you will need to complete some formatting changes, which you will receive in a follow up email. A member of our team will be in touch with a set of requests.

Please note that your manuscript will not be scheduled for publication until you have made the required changes, so a swift response is appreciated.

IMPORTANT: The editorial review process is now complete. PLOS will only permit corrections to spelling, formatting or significant scientific errors from this point onwards. Requests for major changes, or any which affect the scientific understanding of your work, will cause delays to the publication date of your manuscript.

If your institution or institutions have a press office, please notify them about your upcoming paper to help maximize its impact. If they'll be preparing press materials, please inform our press team as soon as possible -- no later than 48 hours after receiving the formal acceptance. Your manuscript will remain under strict press embargo until 2 pm Eastern Time on the date of publication. For more information, please contact mentalhealth@plos.org.

Thank you again for supporting Open Access publishing; we are looking forward to publishing your work in PLOS Mental Health.

Best regards,

Karli Montague-Cardoso

Staff Editor

PLOS Mental Health

***********************************************************

Reviewer Comments (if any, and for reference):
